# Supplementary material for: A normative database of A-scan data using the Heidelberg Spectralis Spectral Domain Optical Coherence Tomography machine
Source: PLoS One. 2021 Jul 1;16(7):e0253720. doi: 10.1371/journal.pone.0253720 (PMC8248651; doi:10.1371/journal.pone.0253720)
Supplement: S1 Table — (DOCX) [file pone.0253720.s001.docx]

S1 Table. Female regression coefficient in multivariate analysis controlling for age with peripapillary retinal nerve fibre layer sectoral thickness values as outcome

| Sector | Disc | p |
| --- | --- | --- |
| Total | –0.58076 |  |
| Nasal superior | 2.71149 |  |
| Nasal | –0.18586 |  |
| Nasal inferior | –0.08341 |  |
| Temporal superior | –2.02026 |  |
| Temporal | –0.84995 |  |
| Temporal inferior | –2.00331 |  |

P-values are coded as: ***<0.001, **<0.01 and *<0.05.
