# Supplementary material for: A normative database of A-scan data using the Heidelberg Spectralis Spectral Domain Optical Coherence Tomography machine
Source: PLoS One. 2021 Jul 1;16(7):e0253720. doi: 10.1371/journal.pone.0253720 (PMC8248651; doi:10.1371/journal.pone.0253720)
Supplement: S2 Table — (DOCX) [file pone.0253720.s002.docx]

S2 Table. Mean volumes by gender

| Volume | Male | Female | P |
| --- | --- | --- | --- |
| Retinal | 8.77 (0.401) | 8.56 (0.326) | >0.0001 |
| RNFL | 0.961 (0.122) | 0.957 (0.101) | 0.7943 |
| GCL | 1.11 (0.11) | 1.07 (0.0764) | 0.002346 |
| IPL | 0.903 (0.0801) | 0.879 (0.0622) | 0.01882 |
| Disc RNFL thickness | 99.9 (9.66) | 99.6 (9.31) | 0.8 |
