# Supplementary material for: A normative database of A-scan data using the Heidelberg Spectralis Spectral Domain Optical Coherence Tomography machine
Source: PLoS One. 2021 Jul 1;16(7):e0253720. doi: 10.1371/journal.pone.0253720 (PMC8248651; doi:10.1371/journal.pone.0253720)
Supplement: S3 Table — (DOCX) [file pone.0253720.s003.docx]

S3 Table. Regression analysis of layer thickness (µm) against age (years) and p-value for each macular segment

| Segment | Retina | | RNFL | | GCL | | IPL | |
| --- | --- | --- | --- | --- | --- | --- | --- | --- |
|  | **R** | **p** | **R** | **p** | **R** | **p** | **R** | **p** |
| Volume | –0.2561 | 0.0003 | 0.0658 | 0.3544 | –0.2911 | <0.0001 | –0.3194 | <0.0001 |
| Centre | 0.0089 | 0.9008 | 0.0011 | 0.9875 | –0.1500 | 0.0340 | –0.0585 | 0.4103 |
| Nasal inner | –0.1821 | 0.0098 | –0.0003 | 0.9969 | –0.2821 | 0.0001 | –0.2930 | <0.0001 |
| Nasal outer | –0.3151 | <0.0001 | –0.0574 | 0.4191 | –0.3051 | <0.0001 | –0.3217 | <0.0001 |
| Superior inner | –0.1438 | 0.0421 | 0.0572 | 0.4214 | –0.2954 | <0.0001 | –0.3444 | <0.0001 |
| Superior outer | –0.2466 | 0.0004 | 0.0964 | 0.1743 | –0.2494 | 0.0004 | –0.2873 | <0.0001 |
| Temporal inner | –0.1117 | 0.1152 | 0.1929 | 0.0062 | –0.1847 | 0.0088 | –0.2071 | 0.0033 |
| Temporal outer | –0.1943 | 0.0058 | <0.0001 | 0.3556 | –0.2516 | 0.0003 | –0.2001 | 0.0045 |
| Inferior inner | –0.0532 | 0.4540 | <0.0001 | 0.9996 | –0.2841 | <0.0001 | –0.3179 | <0.0001 |
| Inferior outer | –0.2521 | 0.0003 | 0.0033 | 0.9627 | –0.2233 | 0.0015 | –0.2138 | 0.0024 |
