# Supplementary material for: A normative database of A-scan data using the Heidelberg Spectralis Spectral Domain Optical Coherence Tomography machine
Source: PLoS One. 2021 Jul 1;16(7):e0253720. doi: 10.1371/journal.pone.0253720 (PMC8248651; doi:10.1371/journal.pone.0253720)
Supplement: S4 Table — (DOCX) [file pone.0253720.s004.docx]

S4 Table. Regression analysis of layer thickness (µm) against age (years) and p‑value for each disc segment

| Segment | Disc | |
| --- | --- | --- |
|  | **R** | **p** |
| Total | –0.1050 | 0.139 |
| Nasal superior | –0.1298 | 0.0671 |
| Nasal | 0.0654 | 0.3573 |
| Nasal inferior | –0.0255 | 0.72 |
| Temporal superior | –0.1910 | 0.0067 |
| Temporal | –0.0533 | 0.4539 |
| Temporal inferior | –0.1312 | 0.0642 |
