# Supplementary material for: A normative database of A-scan data using the Heidelberg Spectralis Spectral Domain Optical Coherence Tomography machine
Source: PLoS One. 2021 Jul 1;16(7):e0253720. doi: 10.1371/journal.pone.0253720 (PMC8248651; doi:10.1371/journal.pone.0253720)
Supplement: S5 Table — (DOCX) [file pone.0253720.s005.docx]

S5 Table. Regression analysis of layer thickness (µm) against refractive error (dioptres) and p-value for each macular segment

| Segment | Retina | | RNFL | | GCL | | IPL | |
| --- | --- | --- | --- | --- | --- | --- | --- | --- |
|  | **R** | **p** | **R** | **p** | **R** | **p** | **R** | **p** |
| Volume | -0.0877 | 0.2171 | -0.1290 | 0.0687 | -0.1138 | 0.1088 | -0.1668 | 0.0182 |
| Centre | 0.0942 | 0.1847 | 0.1426 | 0.044 | -0.0509 | 0.4744 | 0.0341 | 0.632 |
| Nasal inner | -0.0475 | 0.5041 | -0.0751 | 0.2905 | -0.1782 | 0.0116 | -0.2154 | 0.0022 |
| Nasal outer | -0.1614 | 0.0225 | -0.2020 | 0.0041 | -0.1478 | 0.0374 | -0.1946 | 0.0058 |
| Superior inner | -0.0205 | 0.7733 | -0.0769 | 0.2794 | -0.2034 | 0.0039 | -0.2205 | 0.0017 |
| Superior outer | -0.0460 | 0.5178 | -0.0228 | 0.7482 | -0.0193 | 0.7857 | -0.0822 | 0.2473 |
| Temporal inner | -0.0339 | 0.6332 | 0.0723 | 0.3089 | -0.1534 | 0.0301 | -0.1755 | 0.0129 |
| Temporal outer | -0.0413 | 0.5616 | 0.1210 | 0.2555 | -0.0638 | 0.3695 | -0.1073 | 0.1306 |
| Inferior inner | -0.1129 | 0.1113 | -0.1314 | 0.0637 | -0.2102 | 0.3695 | -0.2448 | 0.0005 |
| Inferior outer | -0.1045 | 0.1407 | -0.1570 | 0.0264 | -0.0268 | 0.7065 | -0.0390 | 0.5828 |
