# Supplementary material for: A normative database of A-scan data using the Heidelberg Spectralis Spectral Domain Optical Coherence Tomography machine
Source: PLoS One. 2021 Jul 1;16(7):e0253720. doi: 10.1371/journal.pone.0253720 (PMC8248651; doi:10.1371/journal.pone.0253720)
Supplement: S6 Table — (DOCX) [file pone.0253720.s006.docx]

S6 Table. Regression analysis of layer thickness (µm) against refractive error (dioptres) and p‑value for each disc segment

| Segment | Disc | |
| --- | --- | --- |
|  | **R** | **p** |
| Total | 0.0899 | 0.2056 |
| Nasal superior | 0.0383 | 0.5903 |
| Nasal | 0.1863 | 0.0083 |
| Nasal inferior | 0.1924 | 0.0063 |
| Temporal superior | -0.0722 | 0.3096 |
| Temporal | -0.2365 | 0.0007 |
| Temporal inferior | 0.1029 | 0.1472 |
